# A Review of Automated Bioacoustics and General Acoustics Classification Research

**DOI:** 10.3390/s22218361

**Published:** 2022-10-31

**Authors:** Leah Mutanu, Jeet Gohil, Khushi Gupta, Perpetua Wagio, Gerald Kotonya

**Affiliations:** 1 Department of Computing, United States International University Africa, Nairobi P.O. Box 14634-0800, Kenya; 2 Department of Computer Science, Sam Houston State University, Huntsville, TX 77341, USA; 3 School of Computing and Communications, Lancaster University, Lacaster LA1 4WA, UK

**Keywords:** sound classification, bioacoustics, survey, review, acoustic detection, general acoustics

## Abstract

Automated bioacoustics classification has received increasing attention from the research community in recent years due its cross-disciplinary nature and its diverse application. Applications in bioacoustics classification range from smart acoustic sensor networks that investigate the effects of acoustic vocalizations on species to context-aware edge devices that anticipate changes in their environment adapt their sensing and processing accordingly. The research described here is an in-depth survey of the current state of bioacoustics classification and monitoring. The survey examines bioacoustics classification alongside general acoustics to provide a representative picture of the research landscape. The survey reviewed 124 studies spanning eight years of research. The survey identifies the key application areas in bioacoustics research and the techniques used in audio transformation and feature extraction. The survey also examines the classification algorithms used in bioacoustics systems. Lastly, the survey examines current challenges, possible opportunities, and future directions in bioacoustics.

## 1. Introduction

Automatic acoustic classification also referred to as audio or sound classification, involves the detection or recognition of sound using audio informatics for storage and retrieval, and machine learning techniques for autonomous classification [1,2,3,4,5]. Bioacoustics is the branch of acoustics that is concerned with sounds produced by or affecting living organisms. Bioacoustics is often used in acoustic sensing to monitor biodiversity, especially in visually inaccessible areas [6]. Animal acoustic emissions contain species-specific information that reflects the character and behavior of different living organisms [1]. There are three main application areas of bioacoustics [1]. The first focuses on the classification and analysis of sounds vocalized by different animal species. Its primary aim is to identify sounds that characterize species in different behavioral contexts. The second is concerned with integrating sound signals vocalized by animals with behavioral contexts to understand how the sounds affect the behavior and emotions of the receiver. The third explores the production mechanisms used in sound vocalization processes [1]. The survey presented in this paper explores how current research in automated bioacoustics classification differs from traditional acoustic classification with respect to the techniques used and application areas. We use the term “general acoustic studies” to refer to acoustic research whose primary focus is neither living or non-living organisms.

The scope of our survey is limited to studies that use machine learning as the primary tool for automating acoustic classification. The survey is intended to be a representative rather than an exhaustive review of the state of the research. The survey reviewed 124 publications, spanning 21 years, from 2000–2021. Only papers published in the English language were reviewed. To the best of our knowledge, no recent studies have been undertaken to examine the state of research in this important and fast-growing research area.

Our survey highlights the advances in automated bioacoustics classification, but also identifies the challenges and opportunities presented. For example, we note that the automated classification techniques used bioacoustics still lag behind those in general acoustics. A number of machine learning techniques that have been successfully used in general acoustics are yet to be tested in bioacoustics classification.

The survey sought to answer four questions relating to current bioacoustics research:

- RQ1: What are the main application areas?
- RQ2: What sound data processing and classification techniques are used?
- RQ3: How have the applications described in the studies been implemented?
- RQ4: To what extent have previously identified research problems been addressed by current studies?

Our findings show that current research in bioacoustics is mainly concerned with applications that involve species classification while general acoustic research is primarily concerned with identifying suitable machine-learning algorithms for classifying general sounds. The short-term Fourier transformation (STFT) technique was the most popular audio transformation technique for both bioacoustics and general acoustics studies. Although Mel-frequency cepstral coefficients (MFCCs) and feature extraction techniques were popular in both bioacoustics and general acoustics research, linear prediction cepstral coefficients (LPCCs) techniques were more popular in general acoustics. In bioacoustics research, ensemble classification algorithms were more popular while in general acoustic studies, convolutional neural networks (CNN) classifiers were more popular. Only half of the publications surveyed provided the implementation details of their systems (i.e., architectural design and theoretical background). Most general acoustic studies also described the system workflows, unlike bioacoustics studies. All the studies had a strong focus on results.

The rest of this paper is organized as follows; Section 2 provides a brief background on related work. Section 3 describes the methodology used in the review. Section 4 reports on the results of the review. Section 5 provides a summary of automated bioacoustics research and future trends. Section 6 provides some concluding thoughts.

## 2. Related Work

**Figure 1:**
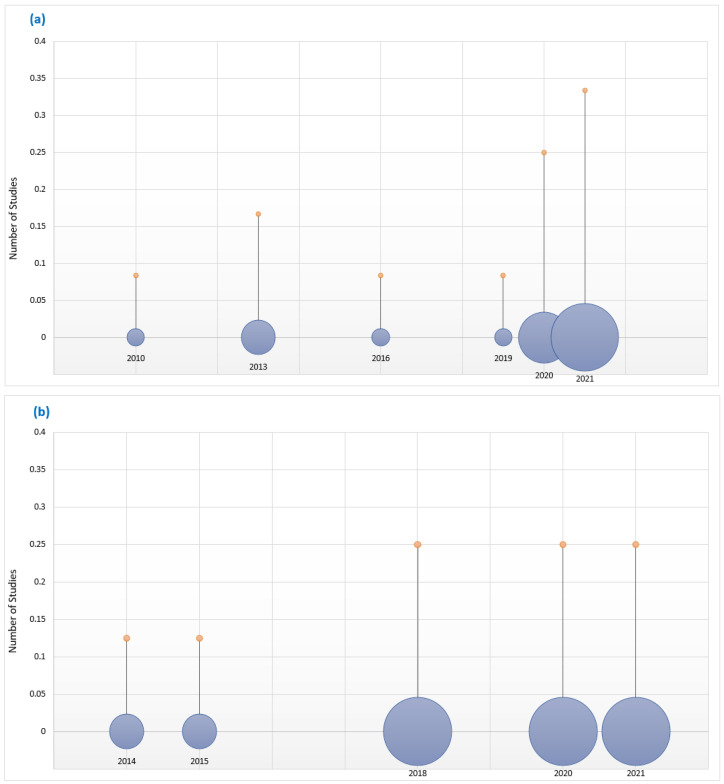
Growth of (**a**) bioacoustics and (**b**) general acoustics research over the years.

This section reviews existing surveys on acoustic classifications to provide the context and motivation for our work. The first survey on bioacoustics sound classification was published in 2010 [7], with the first general acoustics classification survey appearing four years later, in 2014 [8]. Since then, the number of surveys has steadily grown, as shown in Figure 1. The size of the circles indicate the number of surveys published in that year. However, while current surveys suggest significant growth in bioacoustics classification research, many research challenges remain. For example, most surveys focus on well-known taxonomic groups such as birds, and mammals [9] due to the lack of open-source datasets for other species [10]. Secondly, tropical regions are poorly represented in the surveys despite their rich diversity of flora and fauna [11]. Another challenge relates to the running costs of the IoT devices used in data collection. Most of the IoT devices are deployed in remote locations where they are intended to run autonomously for long periods of time, making their operational lifespan crucial in mitigating their running costs. As the devices are battery-operated it is important that effective ways are found improve their energy efficiency. An important aim of our survey was to establish the extent to which the research challenges identified in past surveys have been addressed by current work on acoustics classification.

Current research in acoustics classification spans disciplines such as zoology, engineering, environmental sciences, physics, computer science, and medicine; thus, the range of datasets that we used to source the studies described here vary widely. Out of the 31 survey publications analyzed, twelve focused on bioacoustics sound and the rest on general acoustics. A significant number of bioacoustics survey publications (7) focused on the medical aspects, while general acoustic papers focused on the technology. However, there is growing interest in investigating the technical aspects of bioacoustics classification as highlighted in [9,10,12,13,14,15]. Early reviews [14,15] highlighted Mel-frequency cepstral coefficients (MFCCs) and hidden Markov model (HMM)-based classifiers as the popular acoustic preprocessing and classification techniques. However, recent surveys identify deep learning [13] and ensemble methods as better classification techniques. Other reviews note that widespread use of modern acoustic classification techniques is hindered by the lack of adequate datasets [10] and better de-noising techniques [9,12].

To establish the relevance of existing surveys to our own survey, we conducted a word cloud search to identify comparable surveys. The outcome indicates that the selected surveys used machine learning techniques to identify sounds made by animals. The word cloud search also shows that surveys on bioacoustics monitored biodiversity, characterized vocalizations, or investigated animal behavior. The search shows that the studies in general acoustics surveys focused largely on environmental awareness through sound recognition. The results also show that the selected surveys are relevant and highlights the extent of surveys in acoustic sound classification.

**Figure 2:**
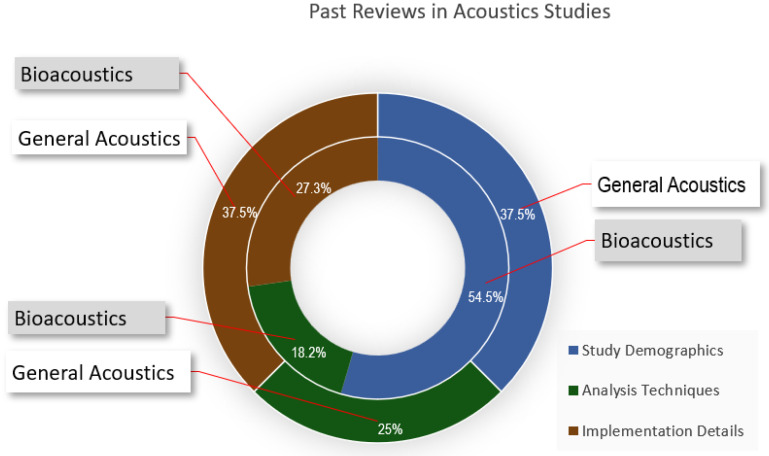
Analysis of previous reviews in acoustics classification.

Further analysis of the surveys revealed that most (55%) of the bioacoustics publications included study demographic information such as year, publisher, and implementation details, as shown in Figure 2. Additionally, most surveys focused on either bioacoustics [1,2,3,4,5,6,7,8,9,10,11,12,16,17,18,19,20,21,22] or general acoustics classification [13,23,24] without direct comparisons. This makes it difficult to share lessons and good practice between the two.

## 3. Methodology

According to [25], reviews with an understanding goal focus more on interpretation than deductive logic. Understanding may be accomplished with the help of two types of reviews; scoping reviews and critical reviews [26]. This review uses a scoping approach where a broader perspective that strives to discern a subject’s overall meaning and relationships is used. The analysis of survey papers consists of six key steps: problem formulation, literature research, screening for inclusion, quality assessment, data extraction, and data analysis and interpretation [26]. The scoping review methodology used in this study excludes quality assessment and therefore uses five of these steps as recommended by [26]. The process is described next.

### 3.1. Problem Formulation

The problem identification process was used to examine related work in past surveys. From this exercise, the research objectives identified were: (i) conducting a comparative analysis of acoustic classification techniques based on their application areas, (ii) highlighting the challenges (gaps) in current research on bioacoustics classification techniques, and (iii) making recommendations for a research agenda for bioacoustics classification techniques based on the application areas.

#### 3.1.1. Literature Search

After examining past reviews, the study mined research papers that addressed the identified research objectives from publications in peer-reviewed research datasets. We screened the relevant papers through an extensive review of literature on the design of bioacoustics and general acoustics classification techniques. This systematic review of the literature used various online databases that index computer science and technology research, namely: IEEE, Science Direct, PubMed, ACM Digital Library, Elsevier, MDPI, Nature, PLOS one, Taylor and Francis, and Springer. The search keywords used were: environmental sound classification, animal sound classification, bioacoustics sound classification, and general acoustics sound classification. To enhance the search process, synonyms complemented some of the keywords. For example, in place of bioacoustics, we also used terms such as animal or bird sounds. Table 1 summarizes the search terms used, the synonyms that complimented them, and the alternative terms used to refine the search.

We reviewed relevant articles published in the past 21 years (2000–2021). This timeframe was selected because practical machine learning techniques started gaining popularity during that time. Only papers written in the English language were included in the review process. The search criteria sought articles that involved sound classification and machine learning technology. Generic search terms (according to the thesaurus of each database) identified the relevant studies. The process of screening relevant studies used the inclusion and exclusion criteria tabulated in Table 2. The identification and elimination of duplicate studies followed. We categorized papers having the same titles or published by the same author on the same subject as duplicates. After the screening and duplicate elimination process, 124 (47 for environmental sound classification and 77 for bioacoustics sound classification) papers emerged as significant for the review.

The initial search process yielded 166 articles (IEEE = 35, Elsevier = 23 Science Direct = 5, ACM Digital Library = 26, MDPI = 17 Springer = 20 and other = 40), with 101 articles for bioacoustics and 65 for general acoustics classification.

#### 3.1.2. Screening for Inclusion

**Figure 3:**
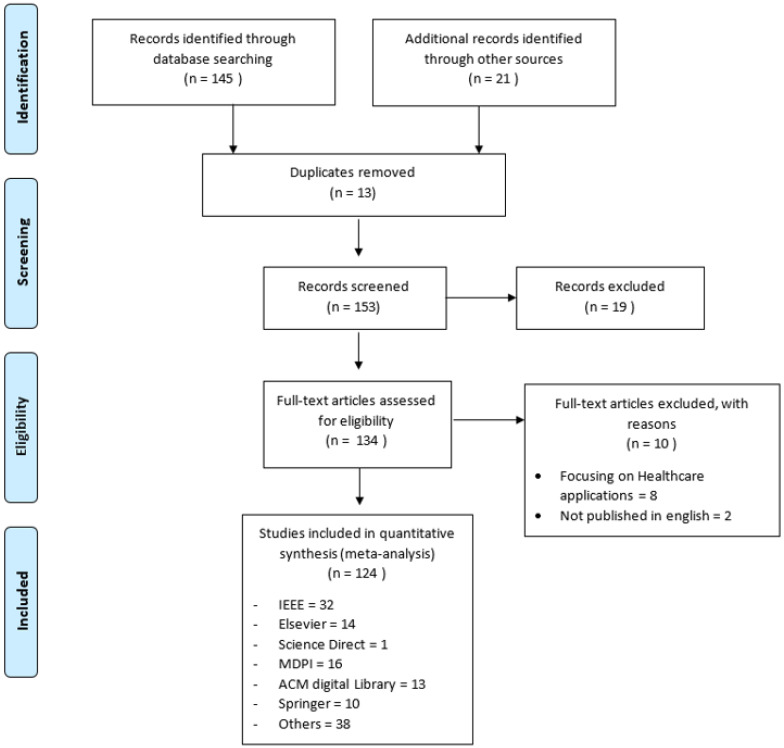
The study selection process.

The Preferred Reporting Items for Systematic Reviews and Meta-Analyses (PRISMA) methodology [27] was used to screen relevant publications on acoustic classification for review. The PRISMA flow diagram in Figure 3 shows the number of papers identified, included, and excluded for the review and the databases used preferred Reporting Items for Systematic Reviews and Meta-Analyses. The identification and elimination of duplicate studies followed the search process. We categorized papers having duplicate titles or published by the same author on the same subject as duplicates. After excluding duplicated papers, 153 articles remained eligible for screening. The screening process resulted in the exclusion of 19 papers that were not in English and those published before 2000, when machine learning technology was still in its infancy. We further excluded ten papers that did not meet the inclusion criteria because they focused on the development of a dataset, monitoring sounds in the music industry or biologically, such as [28,29,30] through a full-text review of the articles.

**Figure 4:**
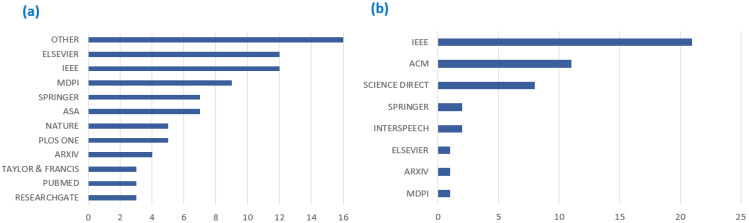
Databases used to retrieve (**a**) bioacoustics and (**b**) general acoustics classification papers.

From this process 124 papers (IEEE = 32, Elsevier = 14 Science Direct = 1, ACM Digital Library = 13, Springer = 10, MDPI = 16, and others = 38), emerged as significant for the final review. Most papers were retrieved from Computer Technology datasets such as Institute of Electrical and Electronics Engineers (IEEE) and the Association for Computing Machinery (ACM) for general acoustics papers. In contrast, bioacoustics papers were common in medical datasets PUBMED and multidisciplinary datasets such as MDPI, as shown in Figure 4. This unsurprising as bioacoustics classification integrates biology and technology disciplines while general acoustics classification focuses largely on technology-related disciplines. Our review used 77 papers representing bioacoustics classification and 47 papers representing general acoustic classification.

#### 3.1.3. Data Extraction

The screened articles were profiled next, in terms of keywords and year of publication to establish the nature and context of the research. The extracted data included: the year of publication, reference, publishers, algorithms used, datasets used, accuracy levels, application area, and the research contribution.

#### 3.1.4. Data Analysis and Interpretation

Following the data extraction stage, research challenges (gaps) were identified using quantitative and qualitative descriptive techniques. Quantitative techniques involved numeric tabulation of observations from the reviews such as the number of datasets or machine learning techniques used in different studies. Qualitative techniques involved description of observations using words such as the limitations identified by previous studies. For example, some studies indicated that there was limited research in tropical geographic areas. These narrations were used to identify and describe the gaps. The results were collated and summarized. The analysis conducted on application areas of bioacoustics versus general acoustics studies provided insights on how research goals between the two areas differed. Additionally, a comparative analysis of acoustics technology revealed how these technologies differ across different application areas. The pre-processing techniques, datasets used, and machine learning algorithms adopted by different studies were tabulated for bioacoustics studies and compared to those used by general acoustic studies. The similarities and differences were documents and used to draw conclusions on preferences for different types of studies. The results of the analysis and interpretations are discussed in the next section.

#### 3.1.5. Publication Demographics

For purposes of this survey, we classified acoustic sound classification publications into two broad categories; those that focused on bioacoustics (where the sound originated from living organisms in the animal kingdom) and general acoustics (where sounds originated from outside the animal kingdom). The word cloud generated from the publication keywords illustrates the relevance of the selected papers. Studies on bioacoustics focused mainly on classifying animal sounds such as birds, insects, and whales, while those concerned with general acoustics were mostly environmental sound signals and not specific to particular species. We confined the scope of the survey to studies that used machine-learning algorithms for sound classification. It is worth noting that several studies also used image recognition techniques classify animals [31,32,33,34,35,36,37,38]. Those studies fell outside the scope of this review.

**Figure 5:**
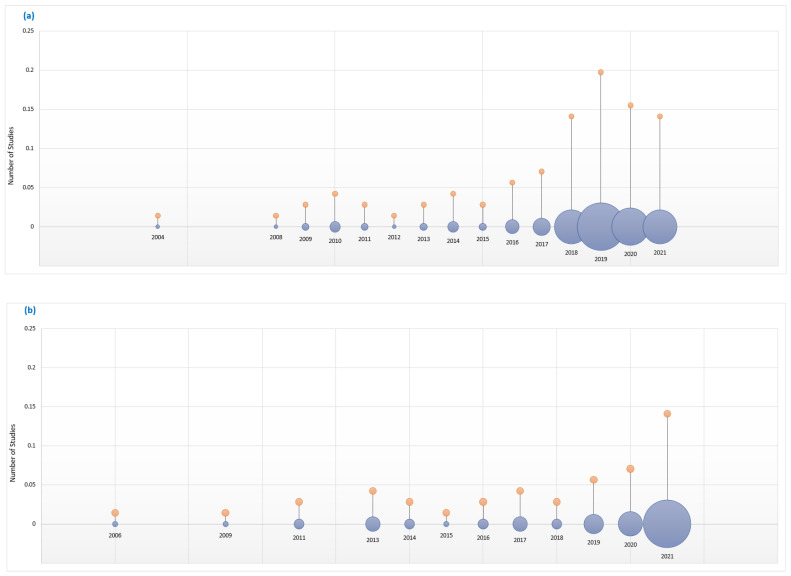
Progress of (**a**) bioacoustics and (**b**) general acoustics research output over the years.

The survey revealed that both categories have received differing attention, with 62.0% of current acoustics classification research focused on bioacoustics and 38.0% on general acoustics, as illustrated in Table 3. This might be explained by the fact that research in bioacoustics classification started earlier than general acoustics research, with bioacoustics research picking up from 2009, as shown in Figure 5, while general acoustics research picked up from 2013. In both cases, the research output has steadily grown. However, the growth of acoustics classification in biology domains has been broader and faster than in technology domains.

## 4. Results

### 4.1. Application Areas

**Figure 6:**
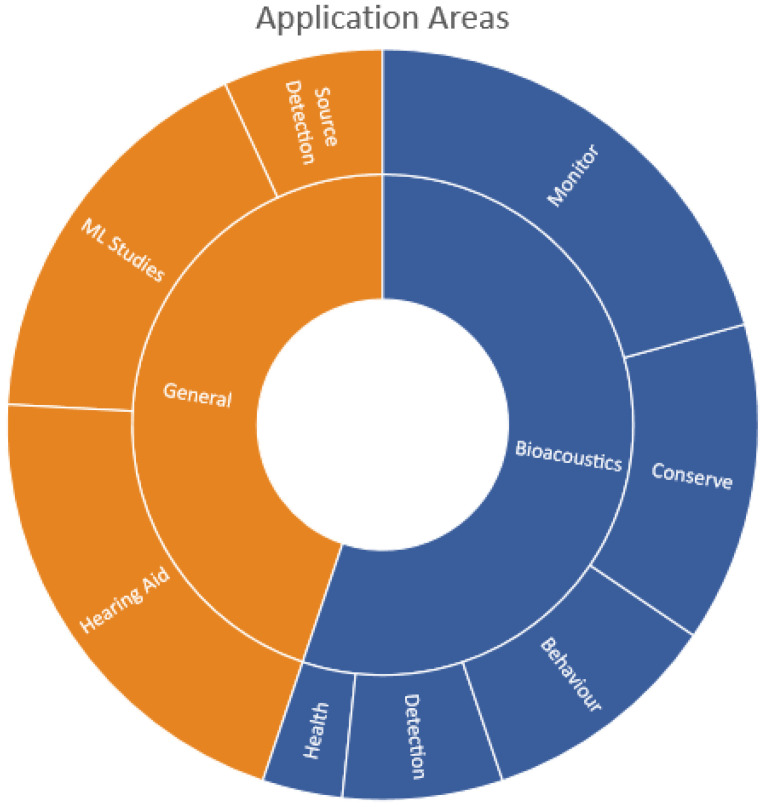
Application areas of research in bioacoustics and general acoustics classifications.

The survey shows that bioacoustics classification has found application in various botany and zoology fields such as: in conserving species [42,43,44,46]; monitoring of inter-species interaction [39,41,49,59,66,69]; understanding animal behavior [56,64,81]; agriculture in pest control [72,74]; and health in detecting sleep disorders [73]. General acoustics classification has applications in: hearing aids [108,109,113,122,124,153]; analyzing machine-learning algorithms [110,111,112,114,154]; or for detecting the sources of sounds [155,156]. Monitoring of species formed the largest bioacoustics application area (84.2%), as shown in Figure 6. Most general acoustics research focused on technology improvement by evaluating machine learning algorithms (38.1%) and detecting the source of the sound through acoustic monitoring (33.3%), as illustrated in Figure 6. A few studies classified environmental sounds to support users with hearing impairments (19%).

**Figure 7:**
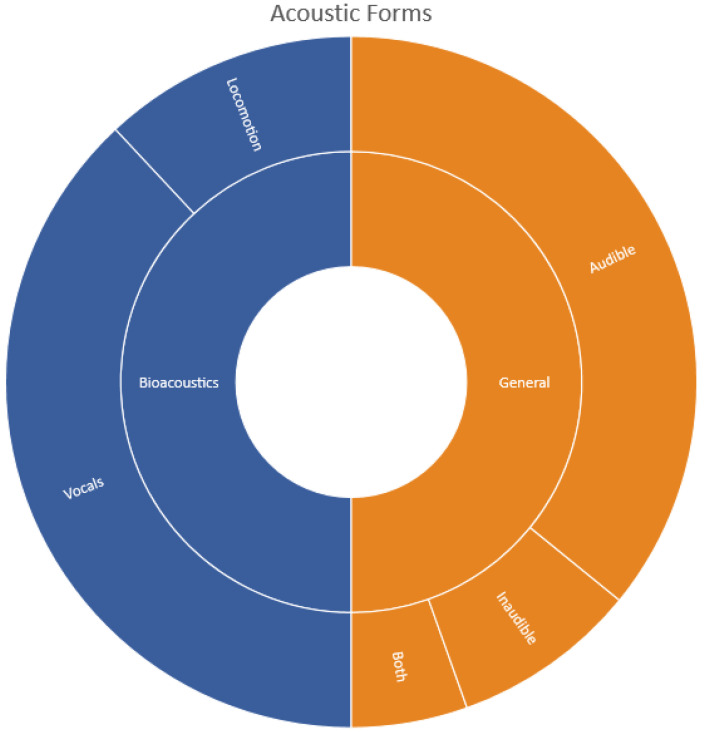
Forms of sound for bioacoustics and general acoustics studies.

Most bioacoustics originated from animal vocals (74%) such as frogs croaking [40,41,42] or birds chirping [9,18,50,58,61] while a few originated from their locomotion (24%) such as bees [56,81,82] or mosquitoes [59] in flight as shown in Figure 7. Insects produce locomotion sounds in five different ways: stridulation, percussion, vibration, tymbal mechanism, or air expulsion [14]. Sounds originating from locomotion are low and sometimes not humanly audible thus, some studies have focused on image recognition to identify insects such as moths [28,33,34,36,38], which can be challenging if the insect is not within the field of vision. It is worth noting that some studies used both image and acoustic classification to classify bird sounds and observed that fusing these approaches achieved the better classification performance [79] compared to individual techniques. Some researchers have also noted that including features that provide visual-based discrimination, extending beyond the bio-acoustically, relevant parameters may offer improved performance [88].

Most publications surveyed (94%) dealt only with acoustic classification for humanly audible sounds. A similar observation was made for general acoustics sounds, where most studies focused on humanly audible sounds such as sounds made by a helicopter, chainsaw, or rain. Limited research existed for non-human inaudible sounds, as seen in Figure 7. This makes it difficult to assess the effectiveness of sound classification techniques for sounds that are not human audible from past studies. Thus, the acoustics research studies reviewed here are biased toward humanly audible sounds.

It is worth noting that the general acoustics research reviewed here was concerned with sounds from both artificial sources, such as car alarms, gunshots, and construction equipment and natural sources, such as rain or animal sounds. Most of the studies examined [112,115,119,144,146] used the two types sound interchangeably, making it difficult to analyze general acoustic techniques exclusively on non-bioacoustics sounds. Establishing whether classification techniques differed for bioacoustics and non-bioacoustics techniques would provide better insight into the factors that influence the choice of classification techniques.

### 4.2. Techniques Used

**Figure 8:**
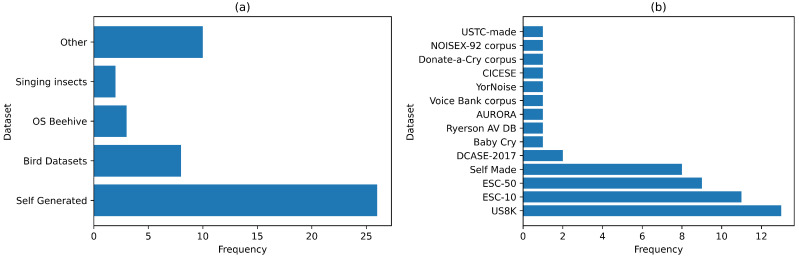
Data sets used for (**a**) bioacoustics and (**b**) general acoustics studies.

Acoustic studies need datasets for training sound classification models. Most of the datasets used for bioacoustics classification are created by the researchers specifically for the study, as shown in Figure 8 [42,45,55,64,72,74]. This was common where publicly available datasets were unavailable. Datasets on insects were few, with the majority having sounds for birds, frogs, cats, whales, and dogs. For general acoustics, the most popular dataset was the US8K (Urban Sound 8K) which contains 8732 labeled sound excerpts of urban sounds [119,127,131,132,138,141,146,149] as shown in Figure 7. The ESC 50 and ESC 10 datasets were also among the popular datasets [119,130,131,132,141,143,144,146,147,148,149]. They contain a mixture of bioacoustics and general acoustics sounds. Most of the past general acoustics studies focused on a mixture of both bioacoustics and non-bioacoustics sounds. Therefore, targeted research is required to examine specific general acoustics based on their application areas.

Audio datasets present several challenges that influence the accuracy of the results obtained. For example, many real-world acoustic analysis problems are characterized by low signal-to-noise ratios and compounded by scarce data [59]. Another challenge is that most large-scale bioacoustics archives contain only a small percentage of animal vocalizations and a large amount of environmental noise, which makes it extremely difficult to retrieve sufficient vocalizations for extended analysis [47]. The majority of the bioacoustics datasets examined had sounds exclusive to certain animal species, rendering them inappropriate for categorizing other different animal species [46]. Several studies also noted that [18] the species belong to specific geographic locations restricting the applications of the datasets. Beehives, for example, are found in various geographic locations with different acoustic backgrounds, and tests should represent each type of background [56]. Typically, locomotion sound falls under two behavioral contexts: (i) sonication (e.g., bees vibrating tomato flowers); and (ii) flight (e.g., bees between tomato flowers). The flight and sonication sound present pronounced differences in acoustic characteristics [82], which should be factored in during classification. A deeper experimental evaluation across multiple datasets is also required to improve the classification performance [107]. These datasets also do not factor in the animal age. Hence another challenge for the classifiers is to discriminate between species regardless of the age or stance [53].

Our survey examined the impact of dataset size and classes on the accuracy obtained from acoustic classification. To achieve this, we assumed that all classes have the same number of instances; hence, we obtained an average of the instances per class. For bioacoustics, the results showed that higher accuracy levels occurred where fewer data (instances) existed, such as using the Cat Sound and Open-Source Beehive project datasets, as shown in Table 4. The number of classes also appeared to impact the accuracy, given that higher accuracy levels occurred where higher instance class ratios existed, as illustrated in Figure 8a.

**Figure 9:**
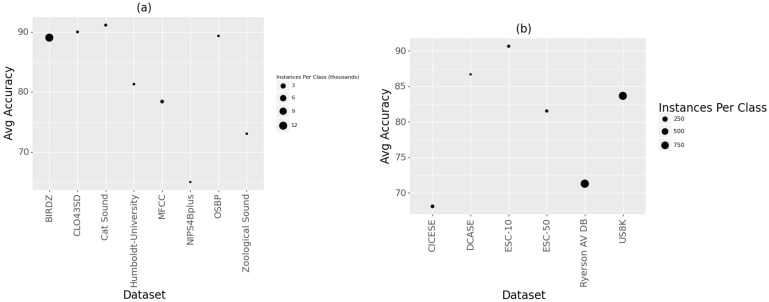
Impact of dataset size on classification accuracy for (**a**) bioacoustics and (**b**) general acoustics studies.

For general acoustics, the results showed that higher accuracy levels were obtained where fewer data (instances) existed, such as using the ESC-10 and DCASE datasets, as shown in Table 5. This is similar to the observations made for bioacoustics. However, the higher the number of classes, the higher the accuracy levels obtained, given that higher accuracy levels occurred where lower instance class ratios existed, as illustrated in Figure 9b. While these results point towards the number of classes having opposite impacts on the results’ accuracy, it is difficult to verify them conclusively because existing studies used only a single dataset. Most studies investigated how the type of algorithm influences the accuracy of the classification process. More research is required to investigate how other factors, such as the size or type of dataset, influence the accuracy of the classification process.

#### 4.2.1. Data Preprocessing

**Figure 10:**
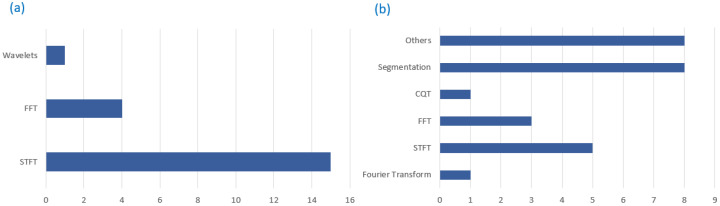
Audio pre-processing techniques used in (**a**) bioacoustics and (**b**) general acoustics studies.

After collecting audio data, they need to undergo preprocessing techniques that clean and transform them for classification. Most bioacoustics [48,49,50,51,53,54,61,62,63,64,68,69,70,71,72,73,74,75,81,82,86], and general acoustic [113,114,115,117,118,120,121,123,124,126,132,135,137,138,139,140,141,142,143,144,145,146,147,149,153,154] studies did not describe the preprocessing techniques that they used. An analysis of the studies that mentioned preprocessing revealed the most popular audio transformation technique as STFT (short-time Fourier transform) among both the bioacoustics [52,60,65,83] and general acoustic [111,119,130] studies (Figure 10). STFT is a powerful general-purpose tool for audio signal preprocessing [157,158,159] where a signal is broken into several signals of shorter duration and then transformed into frequency domains. The other popular technique mentioned was constant-Q transform (CQT) which was used in both bioacoustics [79] and general acoustic studies [148]. It transforms a data series into a frequency domain. The fast Fourier transform (FTT) was popular in bioacoustics studies [47,67]. It expands signals in terms of sinusoids. Both bioacoustics and general acoustic studies employed segmentation [14,39,40,41,42,43,46,83,84,110] to distinguish the sound in question from other sounds such as speech, music, environmental sounds, silence, and combinations of these sounds by automatically revealing semantically meaningful temporal segments in an audio signal [160].

**Figure 11:**
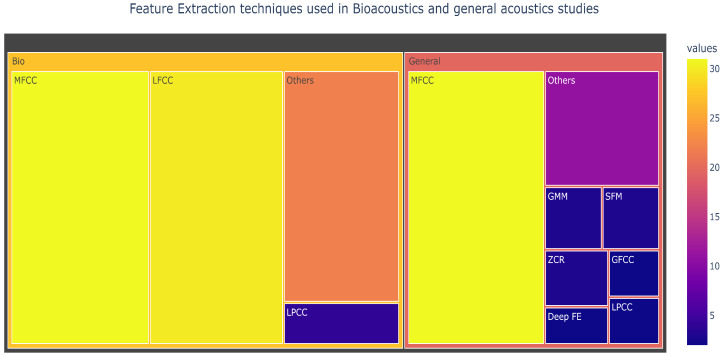
Feature extraction techniques used in bioacoustics and general acoustics studies.

Feature extraction helps derive the audio’s short-time energy, zero-crossing rate, and bandwidth, among other useful features when classifying sound. It reduces the dimension of an audio input vector while retaining the important discriminating feature of the audio. This study revealed that the most popular feature extraction technique uses the cepstral coefficient, as illustrated in Figure 11. Mel frequency cepstral coefficients (MFCCs) use the MEL scale to divide the frequency band into sub-bands and then extract the Cepstral Coefficients using a discrete cosine transform (DCT). The MEL scale is based on how humans distinguish between frequencies, making it a very effective approach for processing sounds. Before the introduction of MFCCs, linear prediction coefficients (LPCs) and linear prediction cepstral coefficients (LPCCs) were the primary feature type for automatic speech recognition, especially with hidden Markov model (HMM) classifiers.

The review observed that MFCCs was popular among bioacoustics studies [39,40,43,44,49,53,61,73,81,82,83,84,86] and general acoustic studies [112,116,125,133,141,148]. Linear frequency cepstral coefficients (LFCC) were popular among general acoustics studies [83,109,110,112] but found fewer applications in bioacoustics studies [14]. Few studies used LPCC [74,127] although it was used in both bioacoustics and general acoustics studies.

#### 4.2.2. Machine Learning Algorithms

**Figure 12:**
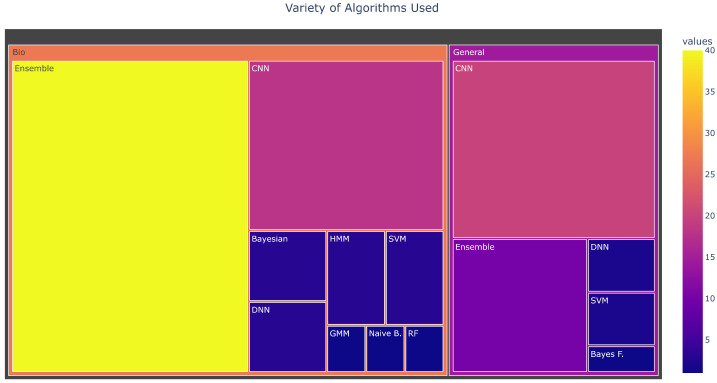
Classification algorithms used for bioacoustics and general acoustics studies.

Audio, sound, or acoustics classification is the process of analyzing audio recordings to identify their origin, type, or environment. The process is often automated using machine learning classification algorithms. Our survey showed that ensemble approaches are the most popular machine learning algorithms used in bioacoustics classification [39,40,43,44,45,48,50,51,53,56,76,77,79,81,82,83,84,86]. Convolutional neural networks (CNN) were the most popular algorithms for general acoustic classifications [113,114,119,121,125,133,136,137,139,141,144,146,148] as seen in Figure 12. The choice of particular classifiers was motivated by the performance of similar classification tasks from previous studies [110,111] or from experiments conducted to identify the most accurate algorithm [113,114]. Some studies did not specify the type of neural network they used; hence we classified them as DNN (Deep Neural Networks) [81,115,124,131,138,154].

**Figure 13:**
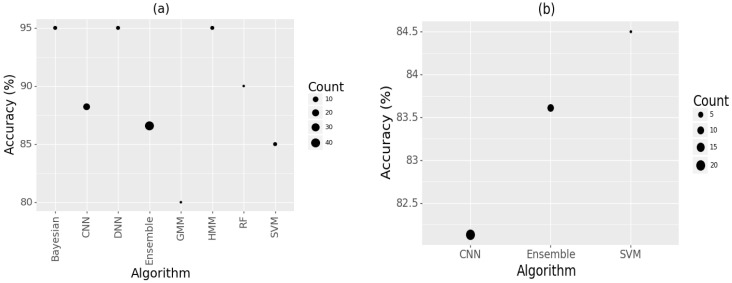
Classification algorithms used for (**a**) bioacoustics and (**b**) general acoustics studies.

Bayesian [58] and hidden Markov models [47] showed the best accuracy levels (based on the figures provided by the authors of these studies) for bioacoustics sounds. However, only a few studies used them, as seen in Figure 13a, due to (1) their high computational cost and (2) greater statistical expertise required than some other methods. This makes it difficult to generalize their efficacy. CNN algorithms and ensemble approaches were more popular; however, they had slightly lower accuracy (87–88%). Ensemble approaches showed better accuracy for classifying general acoustics than approaches based on CNN. However, only a fewer studies used them (Figure 13a). The SVM algorithm gave very high accuracy levels (84.5%), but was used only in a few studies [106,107], which makes it difficult to generalize. These results also show that CNN (at 88%) algorithms perform marginally better than ensemble (at 87%) approaches in bioacoustics studies. However, despite their popularity, they perform poorly (at 82%) in general acoustics studies compared to ensemble approaches (at 83.6%). Therefore, in general, in acoustic studies, ensemble approaches work better. Ensemble approaches also seem to be better at detecting some animal vocalizations, which might explain their accuracy [72]. For example, it has been shown that certain frog species are easily recognized by specific algorithms [71].

Although more accurate, CNN demands large amounts of labeled raw acoustic data [68]. Learning directly from the raw waveform allows the algorithm to automatically select those elements of the sound that are best suited for the task, bypassing the onerous task of selecting feature extraction techniques and reducing possible biases [58]. However, due to the limited datasets available, solutions that yield effective classification results, even when only a small number of per-class training examples are available, should be explored [63]. For example, Ref. [64] proposes a deep learning approach that computes a perceptual embedding of animal vocalizations based on similarity judgments instead of class-specific labels. Similarly, a different study [80] combined transfer learning of a pre-trained deep convolutional neural network (CNN) model and a semi-supervised pseudo-labeling method with a custom loss function to address this challenge. They employ techniques to deal with the lack of class-labeled data, such as transfer learning from a (Multi-Dimensional Scaling) MDS space, attention pooling, and dynamic triplet loss. Combined with the ensemble approach, such techniques have produced better accuracy results [75].

Most acoustic studies did not address resource utilization as part of the algorithm’s efficiency in terms of power and space. Hence, these approaches are unsuitable for real-time resource-constrained applications [76]. Most acoustic presentation approaches require extracting a large set of features, which consumes additional storage, processing, and communication resources.

**Figure 14:**
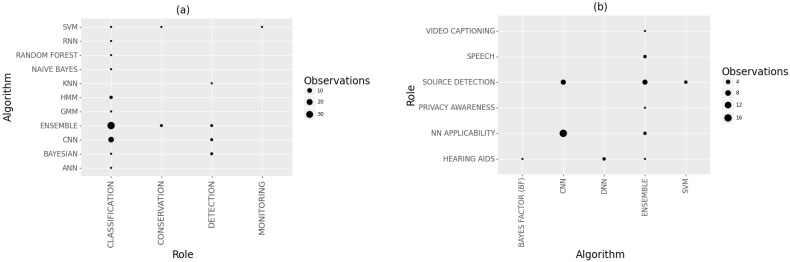
Algorithms used for different acoustic roles in (**a**) bioacoustics and (**b**) general acoustics studies.

The application areas and sources of sound can shed light on the preferred choice of classification techniques to establish the adequacy of an algorithm for a given role. The analysis results shown in Figure 14 reveal that CNN algorithms were predominantly used in general acoustics, where the research investigated ways of enhancing the classification algorithms or detecting the source of the sound. Support Vector Machine (SVM) approaches were also popular for detecting the source of sounds. Other roles, such as speech analysis and video captioning, preferred ensemble approaches. In bioacoustics studies, CNN and ensemble approaches were popular for all roles. However, some algorithms, such as Bayesian approaches were used in species detection.

**Figure 15:**
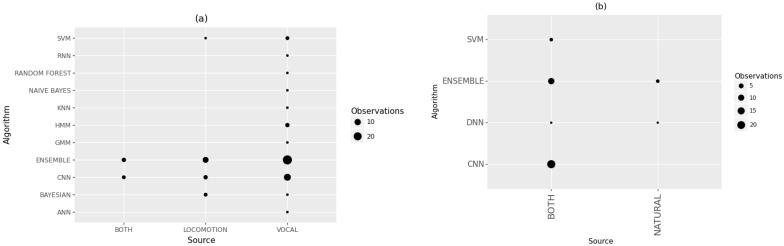
Algorithms used for different sources of sound in (**a**) bioacoustics and (**b**) general acoustics studies.

Both CNN and Ensemble approaches were used to classify natural and artificial sound sources in general acoustic classifications, as shown in Figure 15b. No specific algorithm for natural sound classification was preferred, although such studies avoided CNN and SVM. Studies that investigated bioacoustics preferred CNN and ensemble approaches for analyzing locomotion. However, studies that analyzed vocals also used other algorithms, such as SVM and HMM (Figure 15a). Bayesian approaches were also preferred for analyzing locomotion.

#### 4.2.3. Overtones in Acoustic Techniques

**Figure 16:**
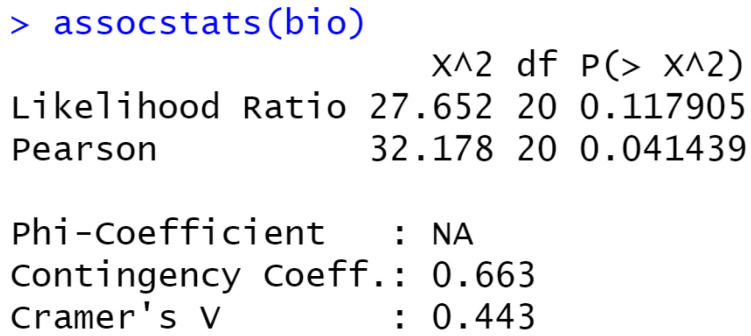
Cramer’s V association test for bioacoustics and general acoustics studies.

Using the R Statistical analysis tool, we used the Cramer’s V method to measure the strength of associations between preprocessing and classification algorithms. The Cramer’s V values for the association between classification algorithms and the preprocessing techniques were obtained as 0.443 and 0.3274 for bioacoustics and general acoustics studies, respectively. While both values indicated a strong association, this value was only statistically significant for bioacoustics studies where the Pearson’s correlation coefficient was 0.0414 (*p* < 0.05), as illustrated in Figure 16.

**Figure 17:**
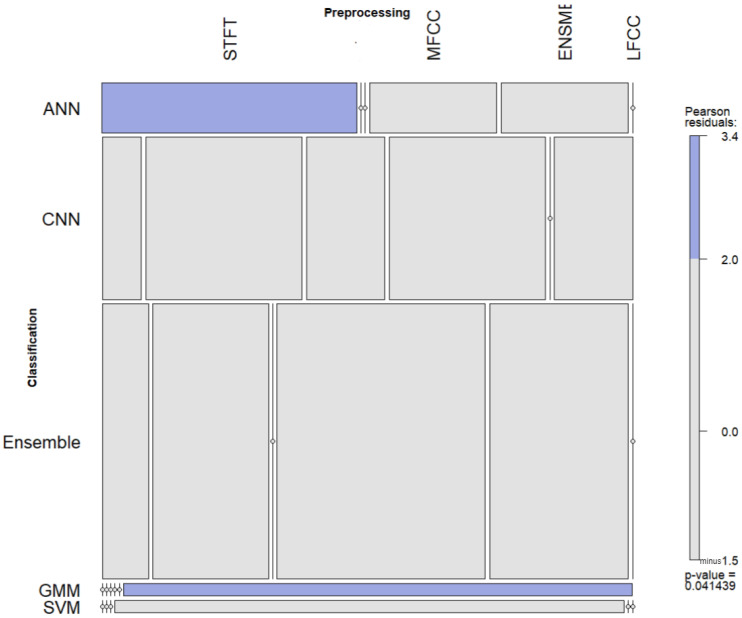
Associations between pre-processing and classification techniques for bioacoustics studies.

Based on these findings, we identified the specific associations for bioacoustics studies using mosaic plots. The blue cells in Figure 17 contribute to the significance of the test of independence, therefore, demonstrating an association between artificial neural networks algorithms for classification and STFT techniques for audio transformation. Similarly, Gaussian mixture model (GMM) classification approaches were strongly associated with LFCC audio transformation techniques. Future studies should seek to understand these associations further through a comparative analysis of different classification algorithms and preprocessing techniques.

**Figure 18:**
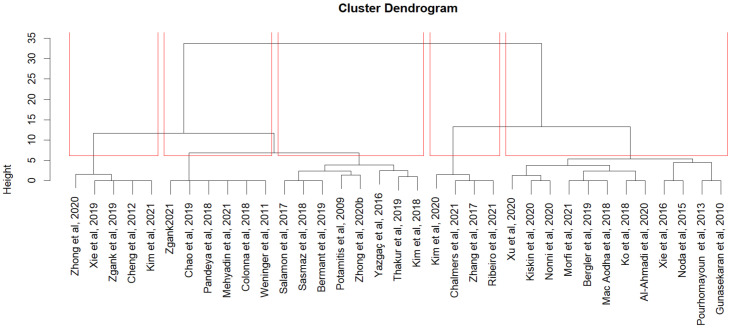
Focus Areas in bioacoustics studies.

To understand how areas of focus varied among the bioacoustics studies that we reviewed, we conducted a cluster analysis of the studies. A cluster analysis groups the observations based on common characteristics to derive further insights from the observations. The results showed that most studies focused on one or two areas. For instance, most studies that examined neural network classifiers did not specify either the audio transformation techniques used (Cluster 5) or the feature extraction techniques (Cluster 3), as shown in Figure 18. Similarly, most of the studies that used ensemble classifiers did not specify the audio transformation techniques, instead they explored either ensemble feature extraction approaches (Cluster 1) or MFCC feature extraction approaches (Cluster 2). Only four studies explored all techniques (Cluster 4). In addition, most studies in Cluster 4 used MFCC and fast Fourier transform (FFT) preprocessing techniques for ensemble classification approaches. It is unclear from our findings how the choice of preprocessing techniques influenced the selection of classification techniques. However, this type of information could benefit other researchers in the field. It would therefore be useful, if studies described the techniques used across all the phases of their bioacoustics classification.

### 4.3. Implementation and Evaluation

**Figure 19:**
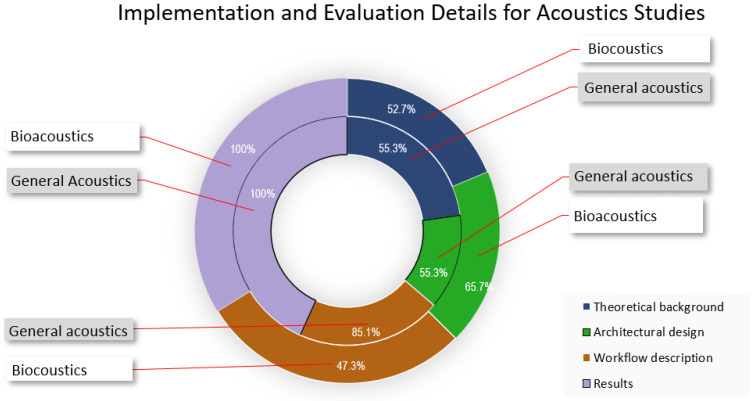
Implementation and evaluation details for acoustic studies.

To understand how the applications identified in the survey were implemented, we examined the theoretical backgrounds, architectural designs, workflow descriptions, and the results presented. A comparison of bioacoustics and general acoustics studies revealed that in both cases, only half of the studies provided theoretical backgrounds or discussed architectural considerations. We attributed this to the fact that the studies prioritized the use of existing technology obtain results at the expense of other considerations. An interesting observation was the emphasis laid on the workflow description by general acoustic studies (85.1%). The results in Figure 19 show that most studies focused on presenting results compared to providing implementation details. The ability to recreate results is a crucial aspect of evaluating the efficacy of any proposed solution, and future studies need to describe implementation as part of the research.

## 5. Discussion and Open Questions

**Figure 20:**
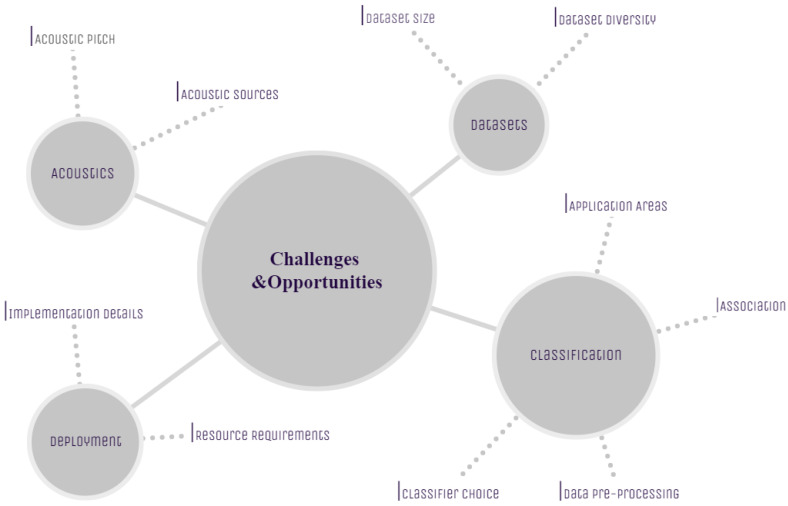
Summary of the challenges and opportunities.

Our survey identified several open questions that might inform future research in bioacoustics. These research gaps are discussed next, and the emerging challenges and opportunities are summarized in Figure 20.

### 5.1. Acoustics

The bioacoustics studies surveyed focused on sounds made vocally rather than through locomotion or other bodily movements. There is need for more research on classifying sounds generated through locomotion and bodily movements. Sonication and isolated motion present pronounced differences in acoustic characteristics, which should be factored in during the classification. Both bioacoustics and general acoustic studies focused on humanly audible sounds, such as those made by frogs or birds, with limited research on less audible sounds made by insects such as moths.

### 5.2. Dataset

Most bioacoustics studies used datasets explicitly generated for the study. Publicly available datasets on insects, arachnids and arthropods were few. The majority of the datasets had sounds for birds, frogs, cats, whales, and dogs. More diverse datasets are needed to enhance research in this area. It is also useful that datasets include not just information on the species, but also geographic locations. Our survey examined the impact of dataset size and classes on the accuracy obtained from acoustic classification. However, it was difficult to verify the findings conclusively as existing studies used only a single dataset. Most studies investigated how the type of algorithm influences the accuracy of the classification process. More research is required to investigate how other factors, such as the size or type of dataset, influence the accuracy of the classification process. A deeper experimental evaluation across multiple datasets is required to enhance the classification performance. Existing datasets also do not factor in the age of the animal, gender, or season.

### 5.3. Classification

While bioacoustics applications in sound detection, species monitoring, and conservation are growing, the volume is still small. The current focus is mainly on classification. The most popular audio transformation and feature extraction techniques among bioacoustics studies were STFT (short-time Fourier transform) and MFCCs. However, few studies have investigated how these techniques’ choices influenced the results’ accuracy. Our survey observed that ensemble approaches were the most popular machine learning algorithms in bioacoustics classification; however, Bayesian and hidden Markov models presented higher accuracy levels. More research is needed on these techniques to generalize their efficacy. There is limited research on how the role or source of sound influence the effectiveness of selected algorithms. Additionally, there is limited understanding of the association between preprocessing techniques and the choice of classification algorithms.

### 5.4. Deployment

Most acoustic studies surveyed did not address resource utilization as part of the algorithm’s efficiency in terms of processing power and memory space requirements. This makes it difficult to gauge their effectiveness for real-time resource-constrained applications. Most studies focused on presenting results compared to providing implementation details such as the theoretical background, architectural and workflow considerations. Further, most of the studies provided more information on feature extraction theoretical backgrounds compared to machine learning. The workflows presented focused more on machine learning compared to feature extraction phases. The ability to recreate results is a crucial aspect of evaluating the efficacy of any proposed solution, and future studies need to adequately describe feature extraction and machine learning implementation aspects as part of the research description.

Classification algorithms present challenges and opportunities for research in new application areas, preprocessing and selection. However, there is also need to investigate and create diverse bioacoustics sources and datasets.

## 6. Conclusions

This survey was a review of acoustic classification techniques based on their application areas to highlight the gaps in existing research on acoustic classification techniques. The results revealed the critical application areas as species classification, done using animal vocals. The popular audio transformation techniques are STFT, while the popular feature extraction techniques are MFCC. The most popular classification approaches are Ensemble and CNN machine learning algorithms. Studies that used ensemble approaches showed a preference for MFCC feature extraction techniques and no specific audio transformation techniques. However, studies that used neural networks showed a preference for LFCC feature extraction techniques and STFT audio transformation techniques. the findings from the survey revealed that most studies focused on disseminating the results rather than implementation considerations. Finally, the study recommended a research agenda for bioacoustics classification techniques.

## Figures and Tables

**Table 1:** Literature search keywords.

Search Key	Acronyms	Search Refinement	Definition
**Bioacoustics **	Animals	birds, wildlife, pests	The branch of acoustics is concerned with sounds produced by or affecting living organisms, especially as relating to communication.
**Non-Bioacoustics**	Environment, artificial		Sounds are produced by artificial sources or both artificial and natural sources.
**Sound**	Noise		Vibrations that travel through the air or another medium, and can be heard when they reach a person’s or animal’s ear.
**Classification**	identification		The action or process of classifying something according to shared qualities or characteristics.
**Technology**	Sensors, Devices		Technology classification of sounds.
**Machine Learning**	Artificial Intelligence	CNN, SVM, Naïve Bayes	The use and development of computer systems that can learn and adapt without following explicit instructions, by using algorithms and statistical models to analyze and draw inferences from patterns in data.

**Table 2:** Literature exclusion and inclusion criteria.

Exclusion Criteria	Inclusion Criteria
**Machine Learning Techniques Based on Images**	bioacoustics classification.
**Research not Published in English**	General acoustic classification.
**Research Published before 2000**	Using machine learning technology
**Sound classification in the medical sector that does not touch on technology**	Peer-reviewed publications
**Papers that were not considered original research, such as letters to the,** **editor comments, etc.**	papers published in English

**Table 3:** Literature exclusion and inclusion criteria.

Bioacoustics Research		General Acoustic Research	
**Citations**	Number	**Citations**	Number
**[14,15,39,40,41,42,43,44,45,46,47,48,49,50,51,52,53,54,55,56,57,58,59,60,61,62,63,64,65,66,67,68,69,70,71,72,73,74,75,76,77,78,79,80,81,82,83,84,85,86,87,88,89,90,91,92,93,94,95,96,97,98,99,100,101,102,103,104,105,106,107]**	77 (62.0%)	**[108,109,110,111,112,113,114,115,116,117,118,119,120,121,122,123,124,125,126,127,128,129,130,131,132,133,134,135,136,137,138,139,140,141,142,143,144,145,146,147,148,149,150,151,152]**	47 (38.0%)

**Table 4:** Bioacoustics dataset size and classification accuracy.

Dataset	Classes	Instances	Ratio	Average Accuracy
**Cat Sound**	2	440	220.00	91.13
**Birdvox70k—CLO43SD**	43	5428	126.20	90.00
**Open Source Beehive Project**	2	78	39.00	89.33
**BIRDZ**	50	602,512	12,050.20	89.04
**Humboldt-University Animal Sound Archive**	2530	120,000	47.40	81.30
**MFCC dataset**	10	7195	719.50	78.40
**Zoological Sound Library**	10,000	240,000	24.00	73.04
**NIPS4Bplus**	87	687	7.90	65.00

**Table 5:** General acoustics dataset size and classification accuracy.

Dataset	Classes	Instances	Ratio	Average Accuracy
**ESC-10**	10	400	40.00	90.66
**DCASE**	16	320	20.00	86.70
**US8K**	10	8732	873.20	83.67
**ESC-50**	50	2000	40.00	81.54
**Ryerson AV DB**	8	7356	919.50	71.30
**CICESE**	20	1367	68.35	68.10

## Data Availability

https://docs.google.com/spreadsheets/d/1Ca-okxXDCsxNt4blr8g1BDXqlxjjV7Cv/edit#gid=1545484044.
